# Treatment with PCSK9 Inhibitors in Patients with Familial Hypercholesterolemia Lowers Plasma Levels of Platelet-Activating Factor and Its Precursors: A Combined Metabolomic and Lipidomic Approach

**DOI:** 10.3390/biomedicines9081073

**Published:** 2021-08-23

**Authors:** Alessandro Di Minno, Roberta Clara Orsini, Mattia Chiesa, Viviana Cavalca, Ilenia Calcaterra, Maria Tripaldella, Andrea Anesi, Susanna Fiorelli, Sonia Eligini, Gualtiero I. Colombo, Elena Tremoli, Benedetta Porro, Matteo Nicola Dario Di Minno

**Affiliations:** 1 Dipartimento di Farmacia, Università degli Studi di Napoli “Federico II”, 80131 Napoli, Italy; 2 CEINGE-Biotecnologie Avanzate, Università degli Studi di Napoli, 80131 Napoli, Italy; 3 Dipartimento di Medicina Clinica e Chirurgia, Università degli Studi di Napoli “Federico II”, 80131 Napoli, Italy; roberta.orsini@alice.it (R.C.O.); ileniacalcaterra@hotmail.it (I.C.); mariatripaldella@gmail.com (M.T.); 4 Bioinformatics and Artificial Intelligence Facility, Centro Cardiologico Monzino IRCCS, 38010 Milano, Italy; mattia.chiesa@ccfm.it; 5 Department of Electronics, Information and Biomedical Engineering, Politecnico di Milano, 38010 Milano, Italy; 6 Centro Cardiologico Monzino, IRCCS, 38010 Milano, Italy; viviana.cavalca@ccfm.it (V.C.); susanna.fiorelli@ccfm.it (S.F.); sonia.eligini@ccfm.it (S.E.); gualtiero.colombo@ccfm.it (G.I.C.); elena.tremoli@ccfm.it (E.T.); benedetta.porro@ccfm.it (B.P.); 7 Fondazione Edmund Mach Research and Innovation Centre, Food Quality and Nutrition Department, Via E. Mach, 1, 38010 S. Michele all’ Adige, Italy; andrea.anesi@fmach.it; 8 Dipartimento di Scienze Mediche Traslazionali, Università degli Studi di Napoli “Federico II”, 80131 Napoli, Italy; dario.diminno@hotmail.it

**Keywords:** PCSK9, untargeted metabolomics, familial hypercholesterolemia

## Abstract

Introduction: Familial hypercholesterolemia (FH) is characterized by extremely high levels of circulating low-density lipoprotein cholesterol (LDL-C) and is caused by mutations of genes involved in LDL-C metabolism, including LDL receptor (LDLR), apolipoprotein B (APOB), or proprotein convertase subtilisin/Kexin type 9 (PCSK9). Accordingly, PCSK9 inhibitors (PCSK9i) are effective in LDL-C reduction. However, no data are available on the pleiotropic effect of PCSK9i. To this end, we performed an untargeted metabolomics approach to gather a global view on changes in metabolic pathways in patients receiving treatment with PCSK9i. Methods: Twenty-five FH patients starting treatment with PCSK-9i were evaluated by an untargeted metabolomics approach at baseline (before PCSK9i treatment) and after 12 weeks of treatment. Results: All the 25 FH subjects enrolled were on maximal tolerated lipid-lowering therapy prior to study entry. After a 12 week treatment with PCSK9i, we observed an expected significant reduction in LDL-cholesterol levels (from 201.0 ± 69.5 mg/dL to 103.0 ± 58.0 mg/dL, *p* < 0.001). The LDL-C target was achieved in 36% of patients. After peak validation and correction, after 12 weeks of PCSK9i treatment as compared to baseline, we observed increments in creatine (*p*-value = 0.041), indole (*p*-value = 0.045), and indoleacrylic acid (*p*-value= 0.045) concentrations. Conversely, significant decreases in choline (*p*-value = 0.045) and phosphatidylcholine (*p*-value < 0.01) together with a reduction in platelet activating factor (*p*-value = 0.041) were observed. Conclusions: Taking advantage of untargeted metabolomics, we first provided evidence of concomitant reductions in inflammation and platelet activation metabolites in FH patients receiving a 12 week treatment with PCSK9i.

## 1. Introduction

Familial hypercholesterolemia (FH), an autosomal dominant disorder that affects ≈ 1 in 220 individuals globally [1], is characterized by extremely high levels of circulating low-density lipoprotein cholesterol (LDL-C) [2,3], a recognized major risk factor for atherosclerosis development and progression and for coronary artery disease (CAD) [4,5,6,7,8]. FH is caused by mutations of different genes involved in LDL-C metabolism, such as those associated with loss of function of the gene coding for the LDL receptor (LDLR), or of the apolipoprotein B gene (APOB) or those associated with gain of function in the gene proprotein convertase subtilisin/kexin type 9 (PCSK9) [9,10,11]. The PCSK9 protease interacts with the LDLR both intracellularly and on the hepatocytes’ surface, and promotes its degradation, thus causing an increase in circulating levels of LDL-C [12,13,14]. Accordingly, PCSK9 inhibitors (PCSK9i) have proven to be an effective therapeutic approach for the treatment of hypercholesterolemia [15]. Although statins are the primary pharmacological approach for the treatment of hypercholesterolemia [16,17,18], especially in patients with very high LDL-C levels, the LDL-C target level is seldom achieved [19,20]. In these cases, clinical studies have shown that the use of two fully human PCSK9i, Evolocumab and Alirocumab, resulted in a significant reduction in LDL-C levels and atherosclerotic plaque regression [21,22].

Pathophysiological mechanisms leading to atherosclerosis and CAD are multifactorial and dyslipidemia has been associated with decreased endothelial function, increased oxidative stress [23], and with an enhanced platelet activity [24] through the release of pro-inflammatory and mitogenic substances that affect the function of endothelial cells and facilitate leukocyte–endothelial cell interactions [25]. In keeping with this, the pleiotropic effect of statins has been reported to be able to, at least in part, counteract CAD onset through the modulation of vascular reactivity and oxidative stress [26]. In contrast, no data are available on the pleiotropic effect of PCSK9i. To this end, we evaluated changes in the plasma metabolome and lipidome to gather a global view of metabolic pathways and characterize metabolites modified following treatment with PCSK9i in FH subjects.

## 2. Materials and Methods

### 2.1. Study Protocol and Population

Approval of the study protocol was obtained from the Local Ethics Committee of the Federico II University Hospital (protocol 2015/261) and the study has been registered on ClinicalTrials.gov (NCT04313270). Twenty-five consecutive patients with a diagnosis of FH and with levels of LDL-C > the 95th percentile (as compared with a sex- and age-matched general population) were recruited from December 2017 to December 2018 at the Lipid clinic of the Federico II University Hospital [27]. The diagnosis of FH was based on the Dutch Lipid Clinic Network Score, and [28] genetic analysis helped identify major causative mutations of familial hypercholesterolemia, including those related to LDL receptor, apoB, and PCSK-9 genes [28]. The main inclusion criteria were a diagnosis of FH and the eligibility of patients to start treatment with PCSK-9i according to available guidelines [29] and criteria identified by Agenzia Italiana del Farmaco (AIFA). Exclusion criteria were age < 18 years, inability to understand or sign the informed consent, high level of transaminases (>3× upper normal limit), hypertriglyceridemia (>150 mg/dL), end-stage renal failure (filtration rate < 30 mL/min/m^2^), current malignant disease or a diagnosis of malignancy in the 2 years prior to the first visit, previous exposure to PCSK-9i, hypercholesterolemia secondary to other causes (e.g., hypothyroidism, corticosteroids, hormone treatments). All the 25 subjects enrolled were on lipid-lowering therapy prior to study entry. Nineteen patients (76%) were receiving statins, whereas 6 reported statin intolerance and were receiving ezetimibe alone. Among the 19 statin-treated patients, 1 was under simvastatin 40 mg, 3 rosuvastatin 40 mg, 5 rosuvastatin 20 mg, 1 pravastatin 40 mg, 1 fluvastatin 40 mg, 5 atorvastatin 40 mg, and 3 atorvastatin 80 mg. None of the patients were on a target LDL value prior to being enrolled in this study. Additionally, as many as 9/25 of these patients (36%) were under antiplatelet drugs, 2 for primary cardiovascular prevention, and 7 for a history of vascular events (coronary artery disease in 6 cases and ischemic stroke in 1 case). To achieve the LDL target, participants involved in the study supplemented the ongoing maximal tolerated lipid lowering therapy with PCSK-9i (Evolocumab^®^ 140 mg every 2 weeks, subcutaneous injection).

A detailed medical history and informed consent were obtained from all patients included in the study. Patient information about age, gender, previous and/or current medical conditions, current and past lipid lowering therapy, and vascular risk factors were recorded at the time of inclusion. Body mass index was calculated as body weight-height^2^. Plasma samples were collected before starting treatment (T0) and after 12 weeks of treatment with PCSK9i (T12).

### 2.2. Biochemical Examination

Total cholesterol (TC) and triglycerides (TG) concentrations, LDL-C and HDL cholesterol (HDL-C), small-dense LDL (sd-LDL), serum creatinine, aspartate aminotransferase (AST), alanine aminotransferase (ALT), creatine phosphokinase (CPK), and plasma glucose were measured employing standard methods before starting treatment (T0), and after 12 weeks of treatment with PCSK9i (T12). LDL particles separation was performed by an electrophoretic Lipoprint System (Quantimetrix Inc., Redondo Beach, CA, USA) [30]. The proportion of sd-LDL particles (subfractions 3–7) to the whole LDL area (subfractions 1–7) was calculated and reported as LDL score, with higher score values reflecting a higher sd-LDL particles content [30]. The mean LDL particle diameter was confirmed (areas under the curve) based on the different electrophoretic mobilities of the 7 LDL species [30].

### 2.3. Untargeted LC-MS Metabolite Profiles

From each patient and at each time point of the study, blood was collected in EDTA and immediately centrifuged at 600× *g* for 15 min to obtain plasma samples that were aliquoted and stored at −80 °C until analyzed. All the samples were thawed on ice before analysis, and 100 μL aliquots of each of them were added to a reference standard solution, i.e., acetonitrile:methanol (50:50 *v*/*v*) solution containing reserpine, 3-nitro-L-tyrosine-13C₉ (3-nitro-tyr-13C₉), L-4-tyrosine-13C₉ (L-4-tyr-13C₉), and trimethylamine-N-oxide (TMAO-d₉) at a final concentration of 4 ng/μL; rivastigmine, acetylsalicylic acid-d₄ (ASA-d₄), prostaglandin F2α-d₄ (PGF2α-d₄), 8-hydroxy-2-deoxyguanosine-15N₅ (8-OHdG-15N₅), and 11-dehydro-thromboxane B2-d₄ (11-DH-TXB2-d₄) at final a final concentration of 2 ng/μL; 12-hydroxyeicosatetraenoic acid-d₈ (12-HETE-d₈) 1 ng/μL and methionine C13 0.8 ng/μL. Protein precipitation was achieved by adding to each sample 300 µL of a precooled acetonitrile:methanol (50:50 *v*/*v*) solution; the mixture was vortexed for 10 s at room temperature and stored for 20 min at −20 °C. Samples were then centrifuged at 12,000 RCF (relative centrifugal force) at 4 °C for 20 min and the supernatant was transferred into vials for metabolomics analysis. Ultra-high-performance liquid chromatography analysis was performed by an Agilent 1290 Infinity UHPLC system (Agilent Technologies, Santa Clara, CA, USA) coupled to a quadrupole time-of-flight mass spectrometry detector (Agilent 6550 iFunnel Q-TOF) equipped with a Dual-Jet ESI source (Agilent, Milan, Italy). Two-microliter samples were injected into a ZORBAX Eclipse Plus C18 Rapid Resolution HD Agilent column (2.1 × 150 mm, 1.8 µm) through an autosampler at 4 °C. The column temperature was set at 60 °C and the flow rate was 0.5 mL/min. Metabolite detection was programmed in both positive and negative ion modes. Mobile phases for both analyses were: water:acetonitrile (95:5 *v*/*v*) with 0.1% formic acid (A) and acetonitrile:water (90:10 *v*/*v*) with 0.1% formic acid (B). The UHPLC gradient was programmed as follows: 1 min with 100% A, 2–8 min with 100–20% A, 8–11.5 min with 20–0% A, 11.5–12.5 min with 0% A, and 12.5–13.5 min with 0–100% A. Initial conditions were reached in 1 min and maintained for 10 min to ensure a complete column re-equilibration. The detector operated in full-scan mode, acquiring mass spectra over the *m*/*z* range of 50–1100 Da, with a scan rate of 1.50 spectra/s. The following additional mass spectrometry setting conditions were employed: gas temp., 250 °C; gas flow, 12 L/min; nebulizer, 45 psig; sheath gas temp., 370 °C; sheath gas flow, 11 mL/min; V cap, 3000 V for positive, 4000 V for negative; nozzle voltage, 1000 V; fragmentor, 150 V; skimmer 1, 65 V; octopole RF peak, 750 V. Samples were analyzed in separate runs (positive and negative ionization modes), in a randomized order. Quality control (QC) samples, obtained by mixing 50 μL of each plasma sample subsequently enriched with the reference standard solution, were used to improve the equilibrium at the beginning of analysis (*n* = 8) and at regular time intervals (every eight injections) to monitor system stability and performance. The QC samples mixed with reference standard solution were prepared together with samples according to the same protocol. MS/MS analysis were performed on the QC samples only for significant compounds, using the same chromatographic separation and ionization conditions reported above. Compounds were targeted using their *m*/*z* value (narrow 1.3 Da) and RT (∆RT 0.9 min), and fragmentation was achieved using two fixed collision energies, 10 and 40 eV. The reference ions, continuously infused into the system, were *m*/*z* 121.050873 and *m*/*z* 922.009798 in positive ion mode and *m*/*z* 119.036320 and *m*/*z* 966.000725 in negative ion mode, respectively.

### 2.4. LC-MS Data Collection

A preliminary quality check of the data collected by LC-MS was performed by MassHunter Qualitative Analysis software (Agilent Technologies). The untargeted metabolomics raw data were processed by MassHunter Profinder software (Agilent Technologies) using the ‘Batch Recursive Feature Extraction’ algorithm that allows for cleaning from background noise and unrelated ions. The parameters used for mass extraction were: 500 counts for the peak filter; charge state limited to 2; permitted ion species for positive ion mode: +H, +Na, and +K, and −H, +Cl, and +CH_3_COO for negative ion mode; and neutral loss of water for both ion modes. To further reduce the acquired data size and complexity, a manual feature evaluation was performed by removing redundant and nonspecific information. This resulted in a dataset comprising a total of 1270 features (625 for the positive ion mode and 645 for the negative ion mode).

### 2.5. Statistical Analysis 

Compounds with more than 4 missing values were removed. The remaining missing data were imputed exploiting the ‘rfImpute’ function, embedded in the ‘randomForest’ R package [31]. The QC signal correction and global normalization were sequentially applied by the ‘QC-RLSC’ strategy and the LOESS normalization, respectively [32]. Differential analyses, evaluating the effect of PCSK9 over time, were performed by the ‘limma’ R/Bioconductor package [33]. The Benjamini–Hochberg procedure was used to control for the false discovery rate (FDR). A compound was deemed significant if the FDR-adjusted *p*-value was <0.05. Demographic and clinical features are expressed as mean and standard deviations.

### 2.6. Metabolites Identification 

Identification of the metabolites was performed by searching their measured accurate *m*/*z* values (10 ppm mass error window) against online available databases such as Metlin (http://metlin.scripps.edu) (accessed on 15 July 2021), Kyoto Encyclopedia of Genes and Genomes (http://www.kegg.jp/kegg) (accessed on 15 July 2021), Human Metabolome Database (http://www.hmdb.ca) (accessed on 15 July 2021), and Personal Compound Database and Library (Agilent Technologies). The results were subsequently verified by comparing their acquired MS/MS spectra with those available on different databases. Annotation or identification was determined following the official classification defined by the Metabolomics Standard Initiative.

### 2.7. Untargeted Lipidomic Analysis

One-hundred-microliter aliquots from all plasma samples were thawed on ice and transferred into 10 mL borosilicate tubes. A slightly modified Bligh and Dyer procedure was used for the extraction of lipids [34]. Briefly, 3 mL of chloroform:methanol 2:1 (*v*:*v*) containing 20 mg/L of butylhydroxytoluene (BHT) was added, and samples were vortexed for 10 s and sonicated in ice in an ultrasonic bath for 15 min (Falc, LabService, Milan). One milliliter of deionized water was then added, and samples were vortexed for 10 s and centrifuged at 3500 rpm for 10 min at 4 °C to promote phase separation. The organic phase was collected, and the aqueous phase re-extracted by adding an additional 2 mL of chloroform with 20 mg/L of BHT. The pooled organic phase was brought to dryness in a Speedvac and re-suspended in 300 µL of methanol:chloroform 9:1 (*v*:*v*). Quality control samples were obtained by pooling 50 µL of each sample. Lipids were injected into an Agilent 1290 Infinity UHPLC (Agilent Technologies, Santa Clara, CA, USA) connected with an Agilent 6550 iFunnel Q-TOF mass spectrometer equipped with a Dual-Jet ESI source (Agilent, Milan, Italy). Lipids were separated under reverse-phase conditions on a ZORBAX Eclipse Plus C18 Rapid Resolution HD column (2.1 × 150 mm, 1.8 µm; purchased by Agilent, Milan, Italy) eluted at 0.3 mL/min. Mobile phase A was acetonitrile:water 50:50 (10 mM of ammonium formate, 0.1% formic acid) and mobile phase B was acetonitrile:water:isopropanol 10:2:88 (10 mM of ammonium formate, 0.1% formic acid). The linear gradient, starting at 65%A:35%B, reached 95%B in 20 min and 100%B in 5 min. Final conditions were kept for 2.5 min to ensure the complete elution of nonpolar lipids. Initial conditions were reached in 0.5 min and maintained for 10 min to ensure complete column re-equilibration.

Full scan analysis over *m*/*z* 100–1200 in positive ion mode and over *m*/*z* 50–1200 in negative were carried out at a scan rate of 1.50 spectra/s. To avoid detector saturation, 0.2 µL of lipid extract was injected in positive ion mode and 2 µL in negative ion mode. Additional mass spectrometry settings were gas temp., 230 °C; gas flow, 12 L/min; nebulizer, 35 psig; sheath gas temp., 350 °C; sheath gas flow, 12 mL/min; V cap, 3500 V for positive, 4000 V for negative; nozzle voltage, 1000 V; fragmentor, 150 V; skimmer 1, 65 V; octopole RF peak, 750 V. MS/MS experiments were carried out under the same experimental conditions using a collision energy of 30 V. Samples were randomized prior to injection. QCs were injected six times prior to analysis, every 8 samples, and at the end of the acquisition to detect instrumentation stability. Data were acquired with MassHunter software (version B.07.00; Agilent, Milan, Italy).

### 2.8. Lipid Annotation 

Lipids were annotated according to their *m*/*z* acquired in high-resolution mode, database search (Lipid Maps (www.lipidmaps.org), Human Metabolome Database (www.hmdb.ca), CEU Mass Mediator (www.ceumass.eps.uspceu.es), and Mass Bank (www.massbank.jp)), and after MS/MS experiments in positive and negative ion modes. Lipids were denoted by head group, total fatty acyl carbon atoms, and unsaturation content (e.g., PC 34:1). For ether/vinyl ether species, both species were reported when it was not possible to annotate the correct form (e.g., PC (O-36:3)/(P-36:2)). Raw data were processed by MZmine 2.3 (www.mzmine.github.io). In positive ion mode, phosphatidylcholine (PC) and lyso-PC (LPC), sphingomyelin (SM), diacylglycerol (DAG), triacylglycerol (TAG), and cholesteryl ester (CE) were detected and annotated. Phosphatidylethanolamine (PE) and lyso-PE (LPE), phosphatidylinositol (PI) and lyso-PI (LPI), phosphatidylserine (PS), ceramide (CER), glucosyl/galactosyl-ceramide (HEX-CER), lactosyl-ceramide (LAC-CER), and 3-O-sulfogalactosylceramide (S-HEX-CER) were detected and annotated in negative ion mode. 

For each lipid class, the unsaturation index (UI) was calculated as an estimate of the average number of unsaturation, using the formula:

UIy = [Σ (% area lipidx × number of double bonds lipidx)]/100(1)

where lipidx represents each single molecular species belonging to the y lipid class, and the average chain length (ACL), an estimate of the average length of acyl chains, using the formula:

ACLy = [Σ (% area lipidx × total number of acyl chains − carbon atoms of lipidx)]/100(2)

## 3. Results

### 3.1. Clinical Characteristics 

Out of the 25 FH subjects enrolled (12 females and 13 males, mean age 51.5 ± 14.5 years), 9 (36%) were under antiplatelet drugs, 2 for primary cardiovascular prevention, and 7 for a history of cardiovascular events (coronary artery disease in 6 cases and ischemic stroke in 1 case). The mean Dutch Lipid Clinic Network Score of the whole patient setting was 9.3. Genetic characterization showed that 20 were heterozygotes for the LDLR mutation; 2 were compound heterozygotes for 2 different LDLR mutations, and 1 was a double heterozygote for mutations of LDLR and of PCSK-9. No major LDLR, APOB, or PCSK9 mutations were documented in 2 subjects. All subjects were on lipid-lowering therapy prior to study entry: nineteen patients (76%) were receiving statins, whereas six reported statin intolerance and were receiving ezetimibe alone. Among the 19 statin-treated patients, 1 was under simvastatin 40 mg, 3 rosuvastatin 40 mg, 5 rosuvastatin 20 mg, 1 pravastatin 40 mg, 1 fluvastatin 40 mg, 5 atorvastatin 40 mg, and 3 atorvastatin 80 mg (Table 1).

### 3.2. Effect of PCSK9i Treatment on Lipid Levels in FH Patients

After a 12 week treatment with PCSK9i, there was a significant reduction in the levels of TC (from 279.6 ± 69.9 mg/dL to 181.8 ± 64.8 mg/dL, *p* < 0.001) and LDL-C (from 201.0 ± 69.5 mg/dL to 103.0 ± 58.0 mg/dL, *p* < 0.001). In contrast, a nonsignificant increase in HDL-cholesterol levels was detected (from 52.5 ± 12.9 mg/dL to 55.0 ± 12.6 mg/dL, *p* = 0.065) and TG levels did not change (from 107.5, IQR: 79.3–146.3 mg/dL to 109.0, IQR: 73.5–146.0 mg/dL, *p* = 0.607). Overall, the LDL-C target (<100 mg/dL for FH patients without CV risk factors and <70 mg/dL for subjects at high cardiovascular risk) was achieved by nine subjects (36.0%).

### 3.3. Metabolomics Changes during Treatment with PCSK9i

**Figure 1:**
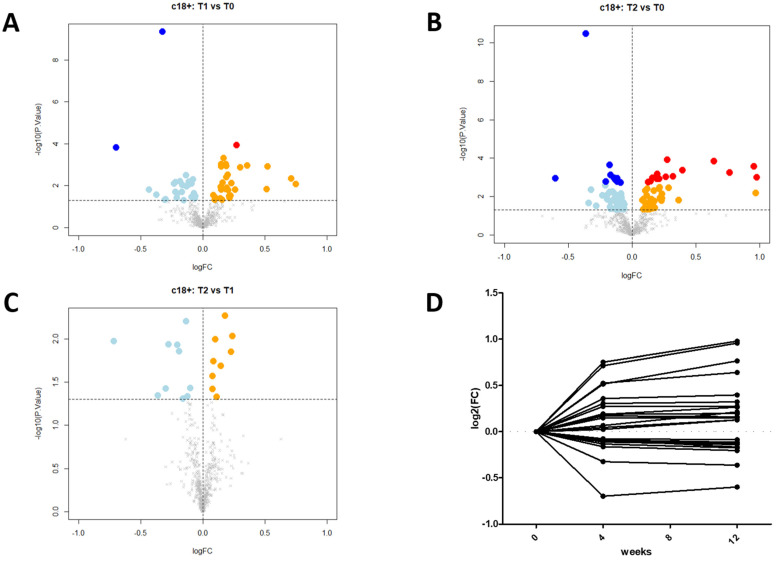
Volcano plots and scatter plot. The results of the statistical analysis for compounds analyzed in positive ion mode are summarized as a volcano plot of log2 fold-change (x-axis) versus − log10 *p*-values (y-axis), for three experimental designs: T1 vs. T0 (**panel A**), T2 vs. T0 (**panel B**), and T2 vs. T1 (**panel C**). Colored dots are the differentially abundant compounds: light blue and dark blue dots refer to compounds downregulated at *p*-value < 0.05 and adjusted *p*-value < 0.05, respectively; orange and red dots are compounds upregulated at *p*-value < 0.05 and adjusted *p*-value < 0.05, respectively. Scatter plot (**panel D**) shows the trend over time of the 19 differentially expressed compounds (adjusted *p*-value < 0.05).

**Figure 2:**
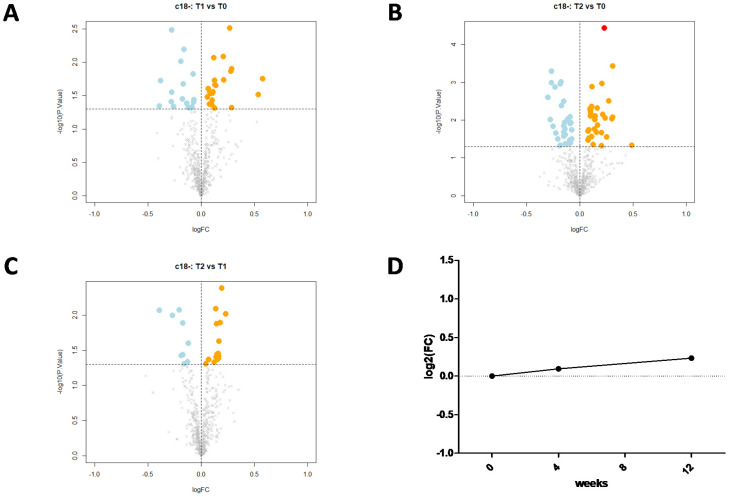
Volcano plots and scatter plot. The statistical analysis results for compounds analyzed in negative ion mode are summarized as a volcano plot of log2 fold-change (x-axis) versus − log10 *p*-values (y-axis), for three experimental designs: T1 vs. T0 (**panel A**), T2 vs. T0 (**panel B**), and T2 vs. T1 (**panel C**). Colored dots are the differentially abundant compounds: light blue and dark blue dots are compounds downregulated at *p*-value < 0.05 and adjusted *p*-value < 0.05, respectively; orange and red dots are compounds upregulated at *p*-value < 0.05 and adjusted *p*-value < 0.05, respectively. Scatter plot (**panel D**) shows the trend over time of 19 differentially expressed compounds (adjusted *p*-value < 0.05). These data refer to the 5 compounds maximally modified after 12 weeks of treatment with Evolocumab^®^, having a Benjamini–Hochberg false discovery rate-corrected *p*-value < 0.05.

Major metabolic changes were detected in the plasma of the FH patients after the 12 week treatment with the PCSK9i. As many as 3300 molecular features were collected in the two different LC–MS modes (1700 in positive and 1600 in negative ionization modes). After visual validation of peak morphology and manual integration, and correction of the whole dataset, a total of 1221 compounds (607 and 614, respectively) emerged as having been affected by PCSK9i treatment. Of them, 97 molecular features showed significant treatment-related changes (ANOVA, *p* < 0.05). Based on the comparison of the *m*/*z* values and the corresponding MS/MS fragmentation spectra against different metabolomics databases, 26 compounds (Table 2) with a log fold-change (FC) ranging from −0.6 to 0.98 showed significant changes after 12 weeks (T12) of treatment (adj. *p* value < 0.05) (Figure 1 and Figure 2). With respect to such compounds, 9/26 were not identified. After adjusting for multiple comparisons, 5/26 metabolites were clearly identified: choline, platelet-activating factor 16 (PAF C16), creatine (Cr), indoleacrylic acid (IA), and indole. Of them (Table 3), both choline (log₂(FC) T2/T0 = −0.113, adj. *p*-value = 0.045) and platelet-activating factor 16 (PAF C16) (log₂(FC) T2/T0 = −0.139, adj. *p*-value = 0.041) were significantly reduced after a 12 week administration of the PCSK9i. In contrast, creatine (Cr) (log2 fold-change T2/T0 = 0.214, adj. *p*-value = 0.041), indoleacrylic acid (IA) (log2 fold-change T2/T0 = 0.128, adj. *p*-value = 0.045), and indole (log2 fold-change T2/T0 = 0.127, adj. *p*-value = 0.045) significantly increased following PCSK9i treatment. In keeping with this, lipidomic analysis following the administration of PCSK9i showed a significant reduction in PC; of 5 polyunsaturated diacyl PC (PC 34:2, PC 36:2, PC 36:4, PC 37:6, and PC 38:4); of two ether/vinyl ether forms of PC (PC O-32:0) and (O-36:3) (P-36:2), and of an oxidized PC molecule (PC 34:1 (OH), *p* values always <0.01).

The parameters shown include mass, mass error (Δppm), mass-to-charge ratio (*m*/*z*), retention time (Rt), log2 fold-changes (the latter, calculated comparing the levels after 12 weeks of treatment [T2] to those at baseline [LogFC T2/T0]).

These data refer to the 26 compounds potentially modified after 12 weeks of treatment with Evolocumab^®^, having a Benjamini–Hochberg false discovery rate-corrected *p*-value < 0.05. 

The parameters shown include: the ionization mode in the mass spectrometry analysis (Mode), molecular weight (MW), mass-to-charge ratio (*m*/*z*), retention time (Rt), log2 fold-changes (the latter, calculated comparing the levels after 12 weeks of treatment [T2] to those at baseline [LogFC T2/T0]).

## 4. Discussion

By employing untargeted metabolomics analysis, we have found significant reductions in the plasma levels of PAF C16, of PC, and of choline following a 12 week administration of PCSK9i. PC is the most abundant phospholipid species in mammals, accounting for about 50% of total phospholipids. It is usually found in the outer membrane leaflet and exhibits bilayer-forming properties. PE is the second-most abundant phospholipid in mammalian membranes, accounting for 20–30% of total phospholipids [35]. It is usually found in the inner membrane leaflet and is a nonbilayer forming lipid. The PC/PE ratio is important for correct membrane integrity and function [36]. PC is synthetized in the liver and approximately 70% of PC derives from the cytidine-diphosphate-choline: 1,2-diacylglycerol choline phosphotransferase (CDP-choline) enzyme (the Kennedy pathway), the remaining 30% being synthesized via the phosphatidylethanolamine N-methyltransferase (PEMT) pathway [37]. At variance with the PEMT pathway where PC derives from three sequential methylations of ethanolamine and PE consumption [38,39], PC biosynthesis via the Kennedy pathway requires choline [40,41,42,43]. This metabolite was significantly reduced after a 12 week administration of PCSK9i. Neither choline nor PC reductions were associated with significant variations in terms of PE, in this setting. Thus, rather for the PEMT pathway, the combined data argue for the Kennedy pathway as being affected after the administration of PCSK9i. The cellular depletion of PC influences the synthesis and transport of high-density lipoprotein (HDL) from the liver [44]. On the other hand, while further data are needed to elucidate the underlying mechanism(s) of its significant reduction in this setting, it is important to emphasize that choline is involved in lipoprotein metabolism [45]. Choline metabolism is important in the regulation of plasma cholesterol levels [46]; choline deficiency is associated with atheromatous changes in aorta, carotid, and coronary arteries [47]; and choline exclusion from the diet inhibits the assembly and secretion of very-low-density lipoproteins (VLDL) from hepatocytes [48,49]. 

**Figure 3:**
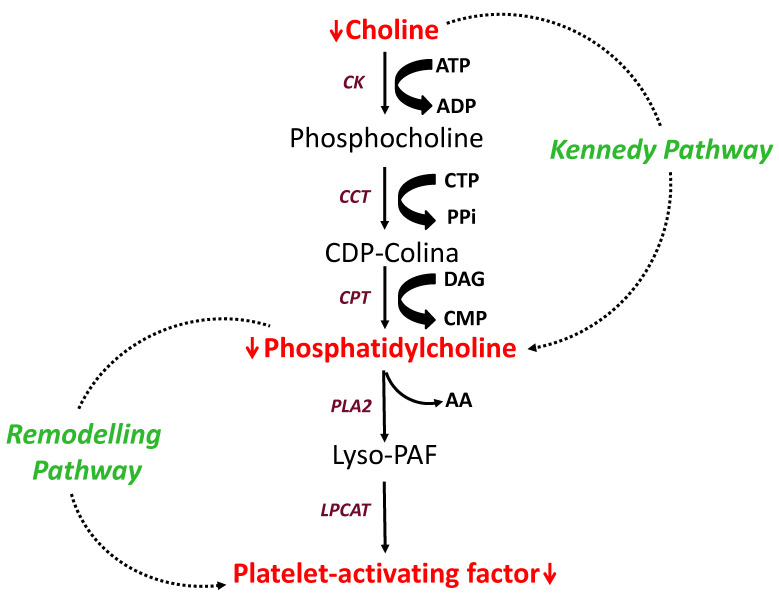
Regulation of Kennedy and remodeling pathways: PCSK9 inhibitor treatment blocks platelet-activating factor biosynthesis by regulating the Kennedy and remodeling pathways. Choline reduction reduces phosphatidylcholine (PC) synthesis, resulting in reduced platelet activating factor (PAF) production. Abbreviations: CK = choline kinase; CTP = cytidine triphosphate; CCT = CTP:phosphocholine cytidylyltransferase; CDP-choline = cytidine diphosphocholine; CPT = CDP-choline:1,2-diacylglycerol choline-phosphotransferase; DAG = diacylglycerol; PLA2 = phospholipase a 2; AA = arachidonic acid; LPCAT = lysophosphatidylcholine acyltransferase. Solid arrow means transformed; red arrow means reduced.

As with choline and PC, PAF was reduced in plasma from FH patients after the 12 week administration of PCSK9i. The multifaceted spectrum of biological and pharmacological effects of PAF, including the ability to influence the function of platelets, confers additional pathophysiological relevance to the present results [50]. Through the remodeling pathway, PC is converted by phospholipase A2 to PAF and lyso-derivatives, both exerting platelet-activating capacities [51] (Figure 3). C16 PAF (platelet-activating factor, 1-O-hexadecyl-2-acetyl-sn-glycero-3-phosphocholine and 1-O-alkyl-2-acetyl-sn-glycero-3-phosphocholine) is a potent mediator of neutrophil migration, and the production of reactive oxygen species and interleukin-6 in human macrophages. In addition to being involved in these well-known events in the initiation and progression of atherosclerosis, PAF plays a key role in platelet function by directly triggering aggregation and potentiating the effects of subthreshold concentrations of other agonists [52,53]. The actions of this agent on the cardiovascular system are mediated by a specific receptor belonging to the seven transmembrane-spanning G-protein-linked receptors family [50]. In the 25 FH subjects examined here, we have already reported a 18% decrease in the urinary excretion of 11-TXB2, a major index of in vivo platelet activation, and a parallel 17% decrease in the urinary excretion of 8-iso-prostaglandin-F2α (8-iso-PGF2α, an established biomarker of lipid peroxidation) after the 12 week administration of PCSK9i [37]. Changes in both analytes correlated with changes in the LDL score. We now show significant reductions in PAF C16 in the plasma samples from these patients collected in parallel with the urine samples used for 11-TXB2 and 8-iso-PGF2α measurements. The relevance of this finding as to: (1) the direct relationship between in vivo platelet activation and circulating plasma levels of PCSK9 [54] and (2) the in vivo reduction in platelet reactivity in hypercholesterolemic subjects on treatment with PCSK9 inhibitors [55] deserves to be evaluated. In the same report [37] on the same population, we have also documented a significant reduction in the lipoprotein Lp(a). By inhibiting the feedback mechanism that accelerates plasmin formation on vascular cells, Lp(a) is involved in atherogenesis, as well as in thrombosis. 

In addition to significant reductions in PAF and its precursors, our untargeted metabolomics approach successfully identified an increase in plasma creatine levels after the 12 week administration of PCSK9i. Anti-inflammatory effects of high concentrations of creatine on endothelial cells have been reported [56]. High creatine levels significantly suppress neutrophil adhesion to endothelium in cell adhesion experiments, and inhibit the expression of adhesion molecules (e.g., ICAM-1, E-selectin) on endothelial cells. Our approach also documents significant increases in the plasma levels of indole and indoleacrylic acid (IA) after a 12 week administration of PCSK9i. Indole and indole derivatives play a key role in the pharmaceutical development of new potent metabolically stable PCSK9 modulators [57,58,59]. Indeed, an indole alkaloid from Nauclea latifolia inhibits PCSK9 and promotes LDL uptake in HepG2 cells. Indole is an intermediate product in the metabolism of tryptophan (TRP) [60,61]. The kynurenine pathway accounts for about ~95% of overall TRP degradation to bioactive catabolites, all being important players in immune response and inflammation [62]. Together with oxidative stress, TRP also exerts key roles in the atherosclerotic process [63,64].

The role of PCSK9 in extrahepatic tissues is little understood so far. Reducing the ability of platelets to oxidize LDL (thus decreasing inflammation-driven platelet-stimulating activity of oxidized LDL) [65], blunting the generation of lipid peroxide-modified phospholipids (a well-known mechanism of platelet activation by dyslipidaemia) [66], reducing lipoprotein (a) levels (a major carrier of oxidized phospholipids) [67], and increasing HDL levels (thus scavenging cholesterol from platelet membranes) [68] are thought to be the underlying mechanisms of the effects of cholesterol esters in thrombosis [45]. Our data argue for events occurring at the level of the PAF activation pathway as helping understand (1) the role of PCSK9 in enhanced in vivo platelet activation, and (2) the prevention of cardiovascular events in hypercholesterolemia by PCSK9 inhibitor administration [69]. 

A major limitation of the present report is the relatively small sample size. FH is an autosomal dominant disorder that globally affects ≈ 1/250 livebirths [1]. To reduce the risk of false positive results, and focus on top differences, only significant changes in lipids classes that discriminate at most the data were considered. The different hormonal status of the enrolled population may play a key role in inflammation and in subclinical atherosclerosis. However, all female patients enrolled (12/25) were on post-menopause and none of them were on hormone replacement treatment.

## 5. Conclusions

In conclusion, taking advantage of untargeted metabolomics, we first provided evidence of concomitant reductions in both inflammation and platelet activation factors in FH patients. These pleiotropic effects could explain, at least in part, the cardiovascular risk reduction and atherosclerotic plaque regression observed following treatment with PCSK9i. The data reported here also support the concept that untargeted metabolomics analysis is a major direction to be pursued to widely investigate treatment effects and explore new clinical applications of such a treatment. Obviously, all biochemical and biological conclusions based on our small sample size need to be confirmed in a larger number of participants with targeted analysis. Nevertheless, the present data provide the rationale for such studies.

## Figures and Tables

**Table 1:** Demographic and baseline characteristics of the FH study population before treatment with PCSK9 inhibitor.

Variable	Study Subjects (n = 25)
Age (years)	51.5 ± 14.5
Age > 60 years	9 (36%)
Male gender	13 (52.0%)
Hypertension	12 (48%)
Cardiovascular events	7(28%)
Obesity	13 (52%)
Diabetes	2 (8%)
Smoking habit	2 (8%)
Body mass index (kg/m^2^)	26 ± 4.8
DUTCH score	9.32 ± 3.64
Alanine aminotransferase (ALT) (IU/L)	24 (IQR:21–26)
Aspartate aminotransferase (AST) (IU/L)	27 (IQR: 21.3- 38.8)
Creatine phosphokinase (IU/L)	110 (IQR: 90–171)
Total cholesterol (mg/dL)	279.6 ± 69.9
Triglycerides (mg/dL)	107.5 (IQR: 79.3–146.3)
HDL-C (mg/dL)	53 ±12.93
LDL-C (mg/dL)	201 ± 69.50
Lp(a) (mg/dL)	69.3(IQR:19.8–83.4)
LDL score	6.6 (IQR:4.65–11.7)
Use of statins	19 (76%)
Ezetimibe alone	6 (24%)
Use of antiplatelets drugs	9 (36%)

Data are presented as number (%) for dichotomous variables, mean ± standard deviation for continuous variables with a normal distribution and median (interquartile range [IQR]) for nonparametric continuous variables.

**Table 2:** Metabolites identified by UHPLC-ESI-QTOF-MS that appeared significantly affected by 12 week treatment with PCSK9 inhibitor.

Mode	MW	*m*/*z*	Rt, min	Adjusted *p*-Value	Log FC (T2/T0)	Putative Annotation
ESI+	428.364	429.372	12.91	0.000	−0.362	Cholesterol derivative
ESI+	173.985	174.993	0.84	0.029	0.276	Not identified
ESI+	183.087	184.095	0.69	0.029	0.641	Drug
ESI+	486.271	487.279	8.91	0.033	−0.174	PA (20:4/2:0)
ESI+	143.095	144.103	0.7	0.033	0.956	Proline betaine
ESI+	627.467	628.475	12.22	0.041	−0.598	Squalamine
ESI+	357.953	358.961	0.84	0.041	0.162	Not identified
ESI+	320.003	321.011	0.84	0.041	0.198	Not identified
ESI+	664.414	665.422	9.31	0.041	0.397	PA (22:6/12:0)
ESI+	133.996	135.004	0.84	0.041	0.196	Not identified
ESI+	301.962	302.97	0.84	0.041	0.323	Not identified
ESI+	523.363	524.371	8.91	0.041	−0.139	PAF C-16
ESI+	293.18	294.188	8.91	0.041	−0.114	Quifenadine
ESI+	165.077	166.085	0.7	0.041	0.978	L-Phenylalanine
ESI+	242.964	243.972	0.84	0.041	0.266	Not identified
ESI+	126.068	127.076	0.7	0.041	0.766	3-heptynoic acid
ESI+	543.33	544.338	7.64	0.041	−0.166	PC (20:4/0:0)
ESI+	131.07	132.077	0.66	0.041	0.214	Creatine
ESI+	523.365	524.373	8.73	0.042	−0.128	PC (0:0/18:0)
ESI+	363.971	364.979	0.84	0.045	0.147	Not identified
ESI+	281.157	282.165	8.91	0.045	−0.087	Xanomeline
ESI+	103.1	104.108	8.91	0.045	−0.113	Choline
ESI+	428.366	429.374	13.09	0.045	−0.204	(3beta,24R,24′R)-fucosterol epoxide
ESI+	187.064	188.072	2.65	0.045	0.128	Indoleacrylic acid
ESI+	117.059	118.067	2.65	0.045	0.127	Not identified
ESI−	351.214	350.206	6.3	0.023	0.233	Not identified

**Table 3:** Identification of compounds that significantly increase (*n* = 3) and decrease (*n* = 2) after 12 weeks of treatment with PCSK9 inhibitor.

Mass	*m*/*z*	RT	Identified Compound	Δppm	Log FC T2/T0
Increased from baseline					
131.07	132.0772	0.66	Creatine	3	LogFC = 0.214
187.064	188.0708	2.652	Indoleacrylic acid	1	LogFC = 0.128
117.059	118.0657	2.652	Indole	5	LogFC = 0.127
Reduced from baseline					
523.363	524.371	8.907	PAF C-16	0	LogFC = −0.139
103.0998	104.1071	8.907	Choline	0	LogFC = −0.113

## Data Availability

In the writing of the manuscript, or in the decision to publish the results.
